# Halo and spillover effect illustrations for selected beneficial medical devices and drugs

**DOI:** 10.1186/s12889-016-3595-7

**Published:** 2016-09-15

**Authors:** Brent D. Kerger, Autumn Bernal, Dennis J. Paustenbach, Gavin Huntley-Fenner

**Affiliations:** 1 Exponent, 320 Goddard, Suite 200, Irvine, CA 92618 USA; 2 Cardno ChemRisk, 130 Vantis, Suite 170, Aliso Viejo, CA 92656 USA; 3 Cardno ChemRisk, 101 2nd St. Suite 700, San Francisco, CA 94105 USA; 4 Huntley-Fenner Advisors, 5319 University Drive, #137, Irvine, CA 92612 USA

**Keywords:** Product recall, Intrauterine device, MoM hip implants, Tysabri, Risk perception

## Abstract

**Background:**

Negative news media reports regarding potential health hazards of implanted medical devices and pharmaceuticals can lead to a ‘negative halo effect,’ a phenomenon whereby judgments about a product or product type can be unconsciously altered even though the scientific support is tenuous. To determine how a ‘negative halo effect’ may impact the rates of use and/or explantation of medical products, we analyzed the occurrence of such an effect on three implanted medical devices and one drug: 1) intrauterine contraceptive devices (IUDs); 2) silicone gel-filled breast implants (SGBI); 3) metal-on-metal hip implants (MoM); and 4) the drug Tysabri.

**Methods:**

Data on IUD use from 1965 to 2008 were gathered from the Department of Health and Human Services Vital and Health Statistics and peer-reviewed publications. Data regarding SGBI implant and explantation rates from 1989 to 2012 were obtained from the Institute of Medicine and the American Society of Plastic Surgeons. MoM implant and explantation data were extracted from the England and Wales National Joint Registry and peer-reviewed publications. Tysabri patient data were reported by Elan Corporation or Biogen Idec Inc. Data trends for all products were compared with historical recall or withdrawal events and discussed in the context of public perceptions following such events.

**Results:**

We found that common factors altered public risk perceptions and patterns of continued use. First, a negative halo effect may be driven by continuing patient anxiety despite positive clinical outcomes. Second, negative reports about one product can spill over to affect the use of dissimilar products in the same category. Third, a negative halo effect on an entire category of medical devices can be sustained regardless of the scientific findings pertaining to safety. Fourth, recovery of a product’s safety reputation and prevalent use may take decades in the U.S., even while these products may exhibit widespread use and good safety records in other countries.

**Conclusions:**

We conclude that the ‘negative halo effect’ associated with a stigma, rather than an objective risk-benefit assessment of medical products can increase negative health outcomes for patients due to reduced or inappropriate product usage.

## Background

When an implanted medical device or drug is withdrawn or recalled, or when negative press reports raise significant concerns about the health risk of that device or drug, multiple impacts, such as market value reductions and reputational declines to a product, brand or manufacturer, may reasonably be expected. For example, at the market level, a broader than expected sell-off of stocks following negative news about a drug or device could be due to the expected loss of revenue, increased manufacturer costs due to litigation or marketing, or an expectation of increased regulation or approval delays. Market level reductions may reflect actual or anticipated changes in patient or consumer attitudes, beliefs, and behavior. At the individual consumer or patient psychological level, for example, perceptions can be influenced by the “halo” effect, a classic finding in psychology wherein a smiling or attractive person is judged to be more honest (positive “halo”), and a non-smiling or unattractive person is judged to be dishonest (negative “halo”) [1]. In general, halo effects are unconscious, are developed on the basis of casual experience rather than through a reasoned process, and lead to unsound judgments [1, 2]. Positive and negative halo effects have been observed in consumer behavior. For example, a product labeled as “organic” may be perceived as higher in fiber and lower in calories or lower in fat (positive halo) [3] and less enjoyable (negative halo) [4]. In the aftermath of negative press reports on medical device or drug withdrawals, individual consumers are subject to such a “negative halo” effect, by which a drug, device, product or product type is then viewed in a negative light across a variety of contexts, only some of which may have been implicated in the recall or withdrawal.

Multiple factors can contribute to the emergence of a negative halo effect, including uncertain scientific findings, negative press reports, and voluntary removal or regulatory restrictions on use of an implant or product. Unsound judgments are bolstered when uncertain scientific findings are taken out of context and/or poorly explained by the news media. Negative press reports about device problems, for example, can create a social context in which fears may become established and flourish [5] and contribute to heightened vigilance of subjective symptoms among some product users or existing implant patients, resulting in a rapid decrease of use or an increase in reported device failures and explantation (removal) procedures. Thus, at the individual consumer level, uncertainty about potentially negative scientific findings or the reasons for a withdrawal, which is fueled by negative media, may result in sales declines for a specific product or a group of products perceived to be similar.

Product-specific recalls and negative reports sometimes result in consumer rejection of an entire class of drugs or implant devices. In the case of heavily publicized events in a drug or device marketplace, a damaged reputation or negative halo of one product can affect all associated products, even if those products were not directly implicated in the original recall or withdrawal event. This phenomenon is considered a “spillover effect.” Spillover effects can be positive (e.g., when competitor products benefit during a recall) or negative (e.g., non-recalled products see declines in sales due to over-generalized concerns) [6]. Negative spillover phenomena are another kind of overgeneralization in which non-implicated products or manufacturers are affected similarly to the directly implicated products [6]. Negative spillover effects can be quite significant, and may even outweigh the benefits that would normally accrue to “surviving” products in the market [6]. Spillover effects may be attributed to psychological or consumer level factors, such as a limited public understanding of functional, clinical, and/or chemical similarity or differences between the recalled drug or device and other products. Positive and negative spillover effects from one product, class of products, or from one manufacturer to another have been observed with many product recalls and withdrawals, including prescription drug withdrawals [6], toy recalls in 2007 [7], and hormone replacement therapy [8].

Positive and negative halo and spillover effects are examples of overgeneralizations. In the case of halo effects, the overgeneralization is from one attribute of a product to other attributes of that same product. In the case of spillover effects, the overgeneralization is from one product to a group of products perceived to be similar (from a lay perspective). In principle, generalization within a class is reasonable and ordinary; however, overgeneralization, when driven by a poor understanding of the scientific evidence or by irrational causes such as fear, may result in unintended negative health effects.

One notable example of a spillover effect with negative consequences involved Vioxx. Early in 2004, Vioxx, manufactured by Merck, was a widely used non-steroidal anti-inflammatory drug with annual sales approaching $2.5 billion. In September, 2004, Vioxx was withdrawn because of a reported association with increased heart attack risk [9]. News about patient deaths was widely publicized, and significant litigation involving Merck ensued, resulting in negative impacts to Merck’s market value [10]. The resulting investigations also led to enhanced warnings on related drugs. Collins et al. [11] reviewed state-level prescription data before, during, and after the Vioxx withdrawal. They reported both positive and negative spillover effects. Patients switched en masse from Vioxx to similar competitive products, non-similar alternative medications, or to no treatment at all, for example. Additionally, a decline occurred in aggregate consumption of non-steroidal anti-inflammatory drugs [11].

Collins et al. [11] argued that this generally rational response to a real or perceived risk may have had inadvertent negative health impacts from a broader public health and well-being perspective. Patient-selected alternatives to Vioxx included over-the-counter analgesics that may not have been the most effective or appropriate alternatives for their condition. Patients who opted to forego medication may have overgeneralized the negative news about Vioxx towards all unrelated non-steroidal anti-inflammatory drugs, and, by going untreated, these patients likely would have experienced worsening health and/or quality of life. Thus, while some patients reduced their exposure to Vioxx-related heart attack risk, the net health and wellness benefit of patient responses in the aggregate was unclear [11]. Excessively broad generalizations of negative news may increase health risk or limit patients’ quality of life (e.g., patients exposing themselves to additional risks through unwarranted explantation surgery, curtailing their purchases of non-recalled pain medications [11], or reducing their preventative health care visits in response to hormone-replacement-therapy warnings [8]).

Patients or end-user consumers are not the only affected persons. Perpetuation of the stigma in the media can influence doctors to offer alternative drugs and medical devices and/or influence patients to choose other medical options, even though these options may be less effective, or may pose significant avoidable health risks. During and after a device recall, some patients are more prone to serious worry about their health status; they may even express their anxiety through litigation and/or selective pursuit of medical providers who can substantiate their fears as a secondary gain [12]. Patient fear may lead doctors to more readily declare device failures, and support patient decisions for explantation based on patient fears in the absence of other implant-related health problems. In essence, anxiety generated by news media and/or uncertain scientific reports (even if not well supported by science or the patient’s clinical status) can become in and of itself a valid medical rationale for implant removal in patients with no clinically apparent failure and no definite implant-related injury or disease. Underlying these behaviors are judgments that are unconscious and resistant to reasoning, new facts, or safety data [1]. Consequently, those stigmatized impressions may persist long after products have been withdrawn or removed from the market, despite the emergence of safety data to the contrary.

Overgeneralizations, such as halo and spillover effects have the potential to more broadly affect public health. They impact the behavior of consumers, patients, and physicians by limiting the use of potentially more efficacious therapies, and by shifting and altering public policy regarding product regulation. The purpose of this study is to explore these effects. Specifically, we review some over-generalization effects of negative information related to selected medical implant devices and one drug between the 1970s and today, including: 1) intrauterine device contraceptives (IUDs); 2) silicone gel-filled breast implants (SGBI); 3) metal-on-metal hip implants (MoM); and 4) Tysabri (natalizumab), a drug for treating Multiple Sclerosis and Crohn’s Disease. The circumstances associated with the stigma and its influence on drug usage and explantation of each device are examined. The impact on public health and the role that each case played in altering public policy are also explored. Recommendations for risk communication to reinforce safe and effective treatments, minimize adverse clinical outcomes, and reduce unintended social impacts are suggested.

## Methods

The medical products included in our analysis were selected based on an initial review of historical drug and device recalls and/or market withdrawal decisions submitted to the U.S. Food and Drug Administration (FDA). Searches were conducted of scientific literature as well as government documents and medical associations for data sources providing insights on the timing and magnitude of impacts of these recall or withdrawal decisions on sales and/or implant removal or replacement of the directly affected product and for similar products. The four medical products/product types chosen were identified based on the availability of: 1) information in the peer-reviewed literature regarding product safety, the product recall, and public perceptions of the product or product type; 2) robust data regarding product usage before and after recall events; and 3) illustrative data and event time lines for examining the severity and duration of negative halo effects on medical products perceived to pose the same or similar risks. Data on public perception or media coverage were not systematically searched for in the popular press. However, peer-reviewed literature and scientific reports were searched and reviewed to add perspective from physicians or mental health professionals who examined or attempted to explain differing perceptions among affected patients surrounding the recall or withdrawal events. Commentaries on patient perceptions that were presented in scientific papers and peer-reviewed literature are noted in Tables 1, 2, 3 and 4 and on selected discussion points.

**Table 1:** Key events surrounding withdrawal of Dalkon Shield IUD

Product	Year	Event
Dalkon Shield IUD	1971	Dalkon Shield IUD introduced on U.S. market
	1972	Public favorable opinion of IUDs peaks (40 %)
	1974	50 % of market share for Dalkon Shield IUD- unique filament design
	1974	Reports of pelvic inflammatory disease (PID), risk of septic abortion
	1974	A.H. Robins Company removes Dalkon Shield from market
	1976	Dalkon Shield sparks FDA medical devices act of 1976
	1970s-1980s	Initial studies: all IUDs increase risk; Later studies: other IUDs safe
	1983	FDA: women should remove Dalkon Shield due to PID
	1985	A.H. Robins Company files for bankruptcy due to class action lawsuit
	1985	Public favorable opinion declines to 20 %
	1986	Other manufacturers pull IUDs from market due to economic reasons
	1994	Public favorable opinion of all IUDs declines to 16 %
	1996	Only 21 % of women associate term *safe* with the use of IUDs
	2000s	Lack of doctors trained in IUD implantation

**Table 2:** Key events surrounding FDA moratorium on SGBI

Product	Year	Event
SGBI	1960s- 1980s	SGBI used for breast augmentation and reconstruction (1 million estimated)
	1976	Amendment to FDA Cosmetic Act: FDA authority to regulate implants
	1980s-1990s	Capsular contracture most commonly reported problem, rupture rate 4-6 %
	1982	Case reports: connective-tissue disease in 3 Australians; lawsuit ensues
	1988	Breast implants- Class III devices: MFs must document safety, efficacy
	1990	Media publicizes concerns, blames FDA for permitting devices
	1991	FDA limits access, MFs must provide *positive demonstration of safety*; FDA acknowledges limited evidence for disease
	1992-1993	>2000 articles published on SGBI-related disease issues; 75 % negative
	1993	AMA: *anxiety is not warranted based on current scientific evidence*
	1994	Removal: augmentation frequencies increased from 9.2 % to 73 %
	1995-1999	Medical Groups: association between SGBI, connective tissue diseases not proven
	2011	FDA concludes SGBI *have reasonable assurance of safety and effectiveness when used as labeled*

**Table 3:** Key events surrounding voluntary recalls of MoM

Product	Year	Event
MoM Hip Implants	1930s	MoM hip implants first used for resurfacing and total replacement
	1950s	MoP implant replaces early gen MoMs, lower early revision rates
	1950s-1970s	MoP revision rates favorable at 5 and 10 yrs. post-implantation
	1970s-1980s	Post ~ 15 years, MoP disintegrates, causes local inflammation, failure
	1990s	New gen MoMs in Europe; early revision rates comparable to MoP
	2003	New MoMs sold in the US
	2003	DePuy ASR XL Acetabular Hip System introduced to Europe
	2005	DePuy ASR XL Acetabular Hip System introduced to US
	2004- 2012	MoM Case reports: inflammation, nervous system and heart effects
	2008	Zimmer Durom AS MoM withdrawn (US)
	2008-2011	Australia and UK hip implant registries: increased rates of early revision for ASR XL Acetabular Hip System
	2008-2011	Reported increased early revision rates (certain MoMs): 3 manufacturers remove devices from markets
	2009	DePuy conducts a voluntary recall in Australia
	2010	Worldwide recall of the DePuy ASR product
	2012	Smith and Nephew withdraw R3 Acetabular System with R3 metal liners (US): reported elevated early revision rates in conjunction with the Birmingham Hip Resurfacing System
	Post-recall	Negative press articles and personal injury litigation ensue in the US

**Table 4:** Key events surrounding withdrawal and reintroduction of Tysabri

Product	Year	Event
Tysabri	2004	FDA approves Tysabri (natalizumab) for relapsing-remitting Multiple Sclerosis (RRMS)
	2004	Biogen reports 3 cases, 1 fatality, of progressive multifocal leukoencephalopathy (PML)
	2005	Tysabri withdrawn from the market
	2006	Clinical trial participants tested for PML; safety data gathered and reviewed by drug sponsor and FDA
	2006	FDA studies evidence, recommends Tysabri reintroduced via restricted distribution program “TOUCH”
	Post-2006	Analyses of TOUCH data, clinical trial data, and continued research help clarify risk of PML; Health care providers have risk model to quantify and rank vulnerability to PML for patients
	Post-2006	Physicians and patients know more about the risk of PML to Tysabri; The number of patients on Tysabri remains well short of expectations.

Data regarding intrauterine device (IUD) use in married contraceptors of 15 to 44 years of age during 1965, 1973, and 1976 was gathered from the Department of Health and Human Services Vital and Health Statistics. The statistics provided in the survey were based on data collected from the National Fertility Study in 1965, and from the National Survey of Family Growth in 1973 and 1976 [13]. Data regarding IUD use for all women, regardless of marital status, were available for 1982, 1995, 2002, and from 2006 until 2008 from the Department of Health and Human Services Vital and Health Statistics [14]. National data on percent contraceptors using the IUD during 1970 and 1988 were reported by Kimble-Haas [15]. Data on IUD use at the Maryland Family Planning Program during 1973, 1977, 1978, 1981, 1983, 1985, 1988-2001 were reported in Cheng [16] and Hubacher [17].

Silicone gel-filled breast implant data from 1989 to 1997, obtained from the American Society of Reconstructive Plastic Surgeons (ASRPS), were reported in the Institute of Medicine’s (IOM) report on the Safety of Silicone Breast Implants [18]. The committee interpolated the reported rates for certain years by assuming that non-survey year rates were at the midpoints between bracketing survey years (e.g., 1990-1992). The IOM (1999) reported that the implant totals from 1989 through 1997 were 130,000, 130,000, 101,000, 53,000, 59,000, 65,000, 87,000, 108,000, and 146,000, respectively (total for the period—879,000), and the explants were 12,000, 12,000, 19,000, 26,000, 32,000, 38,000, 26,000, 14,000, and 14,000, respectively (total for the period—193,000). The American Society of Plastic Surgeons (ASPS) also published data regarding procedures and removals reported by surgeons during 1992, 1994, 1996, and from 2000 to 2012 [19]. The reporting surgeon base reportedly included approximately 23,000 physicians between 2000 and 2012 [20]. However, the surgeon basis reported on prior to 2000 was much smaller, approximately 3000.

Metal-on-metal hip implant use and explantation data were extracted from annual reports available online from the England and Wales National Joint Registry (NJR) [21, 22] and from published scientific articles based on data from the NJR [23]. The United Kingdom Joint registry data was chosen for analysis based on information that the voluntary recalls were based in part on data generated by this registry, and little data available regarding the early revision experience in the U.S.

Tysabri patient data were gathered from Elan Corporation or Biogen Idec Inc. Fourth Quarter Reports for each calendar year and are based on information from the TYSABRI Outreach: Unified Commitment to Health (TOUCH) prescribing program database and other third party sources [24–31]. All data used in this manuscript is openly available from the sources described above.

## Results

### The intrauterine device (IUD)

We identified several key events surrounding the withdrawal of the Dalkon Shield IUD, as well as documentation of patient perceptions regarding IUD use from reports and peer-reviewed studies. Table 1 provides a chronological summary of selected key events and findings relating to the recall of the Dalkon Shield IUD and effects on the IUD market in the U.S.

Beginning in the mid-1960s, the IUD was introduced in the U.S. and was used as a contraceptive product by an increasing number of women [13]. At the time of its introduction to the U.S. market in 1971, the Dalkon Shield did not require FDA approval as a medical device [16] (Table 1). The Dalkon Shield was used by approximately 2 million women by 1974 [16, 32]. Based on reports of IUD use during the early 1970s, we estimated that the Dalkon Shield obtained approximately 50 % of the IUD market in the U.S. [13, 14, 16, 32, 33] (Table 1). Time trend data of U.S. women’s attitudes about contraceptive methods, including IUDs, reflect that favorable opinions on IUD use peaked at approximately 40 % in 1972 [34] (Table 1).

Unlike other IUDs, the Dalkon Shield was designed with a multifilament string, which was ultimately discovered to promote bacterial infections of the uterus, and an increased risk of septic abortion if contraception failed [15, 16, 32]. In 1974, these findings led to the removal of Dalkon Shield IUDs from the market by the manufacturer, A.H. Robins Company [15, 16, 32, 35] (Table 1). Congress then passed the 1976 Medical Devices Amendment, which led to increased FDA regulation and further investigations of all IUDs during the 1970s and 1980s, which reported higher levels of pelvic inflammatory disease (PID) in women [36] (Table 1). In 1983, FDA recommended that women still using Dalkon Shields have them removed because of increases in PID [16] (Table 1). Later studies reported that other IUD designs were not as problematic as the Dalkon Shield [36] (Table 1). Reportedly because of economic strain from increased litigation and, specifically, a class action lawsuit, A.H. Robins Company filed for bankruptcy in 1985 [16, 33] (Table 1). At this time, it was reported that the percentage of women with a favorable opinion toward IUDs decreased to 20 % by 1985 [34] (Table 1). By 1986, only one IUD (Progestasert) remained on the U.S. market, and by 1988, with the introduction of the Paragard IUD, still only 2 % of contraceptors used IUDs [15, 16]. By 1994, public favorable opinion decreased to 16 %, and in 1996, it was reported that only 21 % of the women associated the term “safe” with IUDs [34] (Table 1).

**Fig. 1:**
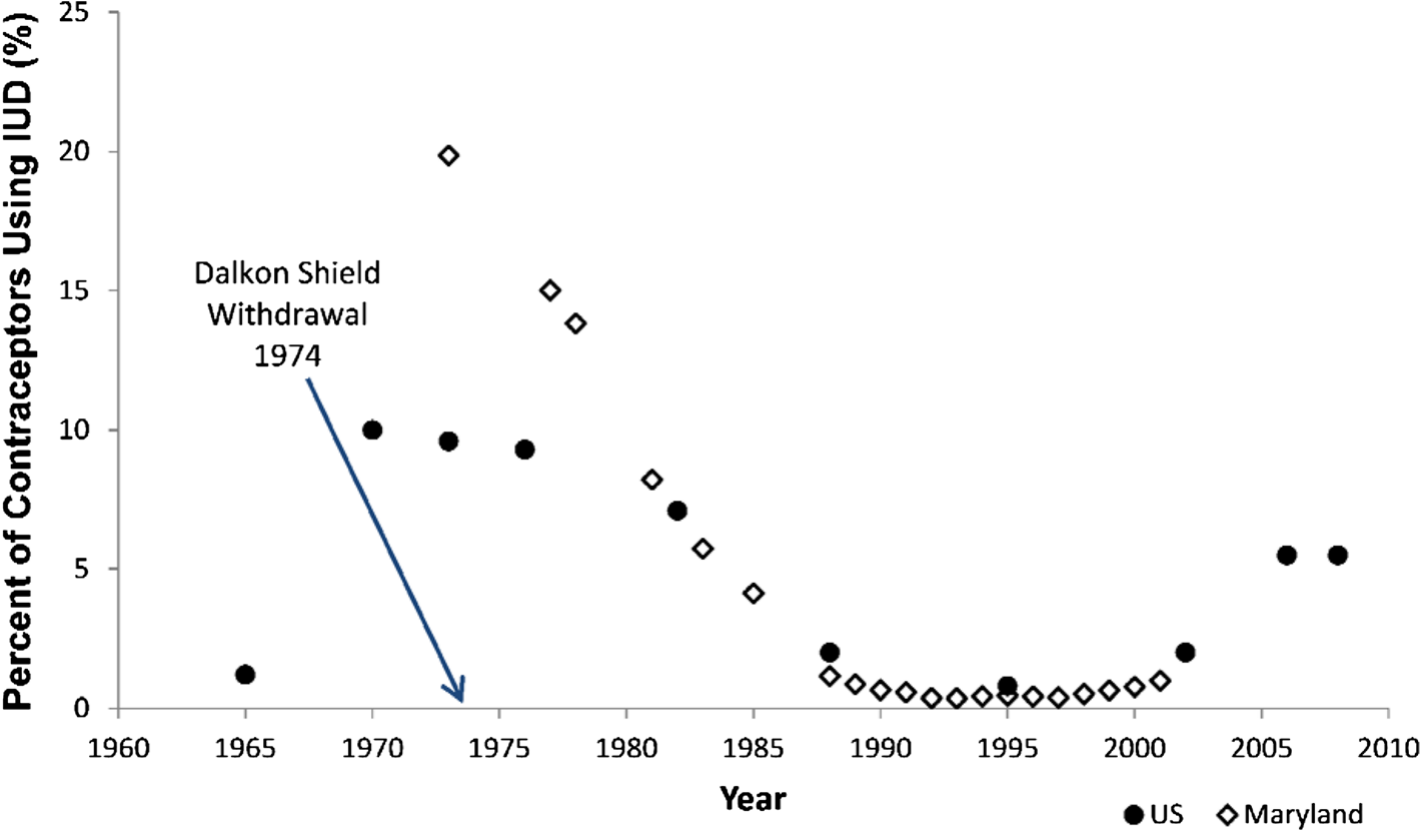
Intrauterine Device (IUD) use, percent of total contraceptors (1965-2008). Closed circles represent the percentage of married contraceptors, 15 to 44 years of age, using an IUD during 1965, 1973, and 1976, as well as for all women, regardless of marital status, during 1970, 1982, 1988, 1995, 2002, 2006, and 2008. Data were obtained from Department of Health and Human Services Vital and Health Statistics as reported by Mosher [13], Mosher et al. [14], and Kimble-Haas [15]. Open circles represent the percentage of women in the Maryland Family Planning Program using IUDs during 1973, 1977-1978, 1981, 1983, 1985, and 1988-2001 as reported by Cheng [16] and Hubacher [17]. The arrow indicates the date at which the Dalkon Shield was voluntarily withdrawn from the market

According to Department of Health and Human Services Vital and Health Statistics, approximately 10 % of U.S. contraceptive users chose the IUD in 1970 (Fig. 1) [16]. After the Dalkon Shield IUD was pulled from the market in 1974, the percentage of IUD users dropped steadily, from 9.3 % in 1976 to 7.1 % in 1982 [13, 14] (Fig. 1). Following FDA’s recommendation in 1983 that remaining users remove their Dalkon Shield device, IUD use among contraceptors fell to 2 % by 1988 as reported by Kimble-Haas [15] (Fig. 1). In 1982, 2.2 million women still used IUDs, and by 1988, only 700,000 women used them [33]. IUD use hit its lowest point in 1995, with only 0.8 % of U.S. contraceptors choosing IUDs [14] (Fig. 1). IUD use in Maryland, as documented in the Maryland Family Planning Program also mirrored the national decline. In 1973, 19.9 % of contraceptors at the Maryland Family Planning Program used IUDs; however, by 1998, only 0.5 % of women used IUDs [16] (Fig. 1). By 2002, the number of contraceptors using IUDs rose back to 2 %, and from 2006 to 2008, rose to 5.5 % of contraceptors in the U.S. [14] (Fig. 1).

### Silicone gel-filled breast implants (SGBI)

We identified several key events surrounding the moratorium on SGBI, as well as documentation of patient perceptions regarding SGBI explantation from reports and peer-reviewed studies. Table 2 provides a chronological summary of selected key events and findings relating to the FDA limitations on use of SGBI and its effects on their explantation and continued use on the medical device market in the U.S.

Since the 1960s, silicone gel-filled breast implants (SGBI) have been used for breast augmentation and reconstruction. One million women were estimated to have undergone breast augmentation surgery between 1963 and 1988 [37] (Table 2). The 1976 Medical Devices Amendment to the Food, Drug, and Cosmetic Act, which had been passed because of negative reports regarding the Dalkon Shield, provided the FDA authority to regulate breast implants. In 1982, case reports described connective-tissue disease in three Australian women with SGBIs, and the first multi-million dollar lawsuit alleging that silicone implants caused systemic disease was filed in California (Table 2). In 1988, breast implants were designated as Class III devices, and manufacturers were required to document their devices’ safety and efficacy [38] (Table 2).

The most commonly reported problem with SGBI was capsular contracture (fibrous tissue capsule formed around the implant as a foreign body response), which Gerszten [39] described as potentially involving “*moderate to extreme hardening of the breast, tightness, mild to severe pain, and deformity or distortion of the breast*”. Diagnosis of the severity of capsular contracture is subjective, and thus reports of frequency/severity are difficult to compare. The reported incidence of contractures ranged from 0.6 % to 100 % depending on the study [39]. In addition to capsular contracture, 4 to 6 % of SGBIs had been reported to rupture based on data reported through the early 1990s [40, 41] (Table 2). It should be noted that Brown et al. [42] later reported much higher rates of implant rupture (e.g., ~70 % overall among asymptomatic women enrolled in the National Cancer Institute study) with significant correlations to implant age, implant type (i.e., double vs. single lumen), implant location (i.e., subglandular vs. submuscular), and implant manufacturer.

In 1990, a CBS television show (“Face to Face with Connie Chung”) reported concerns about breast implants being associated with autoimmune disease, blaming FDA for permitting hazardous medical devices to be sold [43] (Table 2). In late 1991, FDA announced a decision to limit access to SGBIs because many questions remained regarding implant durability/ longevity, rupture rates, and the composition and potential health consequences of leaking silicone gel, asserting that the manufacturers did not provide “*a positive demonstration of safety*” [41]. However, FDA officials acknowledged that “*the link, if any, between these implants and immune-related disorders and other systemic diseases is also unknown*” [41]. It was reported that the occasion of the FDA’s action to limit SGBIs to only breast reconstruction patients (e.g., breast cancer surgery patients) in regulated clinical trials at the National Cancer Institute precipitated an increase in negative press reports about device rupture or leakage and associated health concerns [44]. Between 1992 and 1993, over 2000 news articles were reportedly published on SGBI-related disease issues, of which about 75 % had portrayed SGBI in a negative manner [44] (Table 2).

**Fig. 2:**
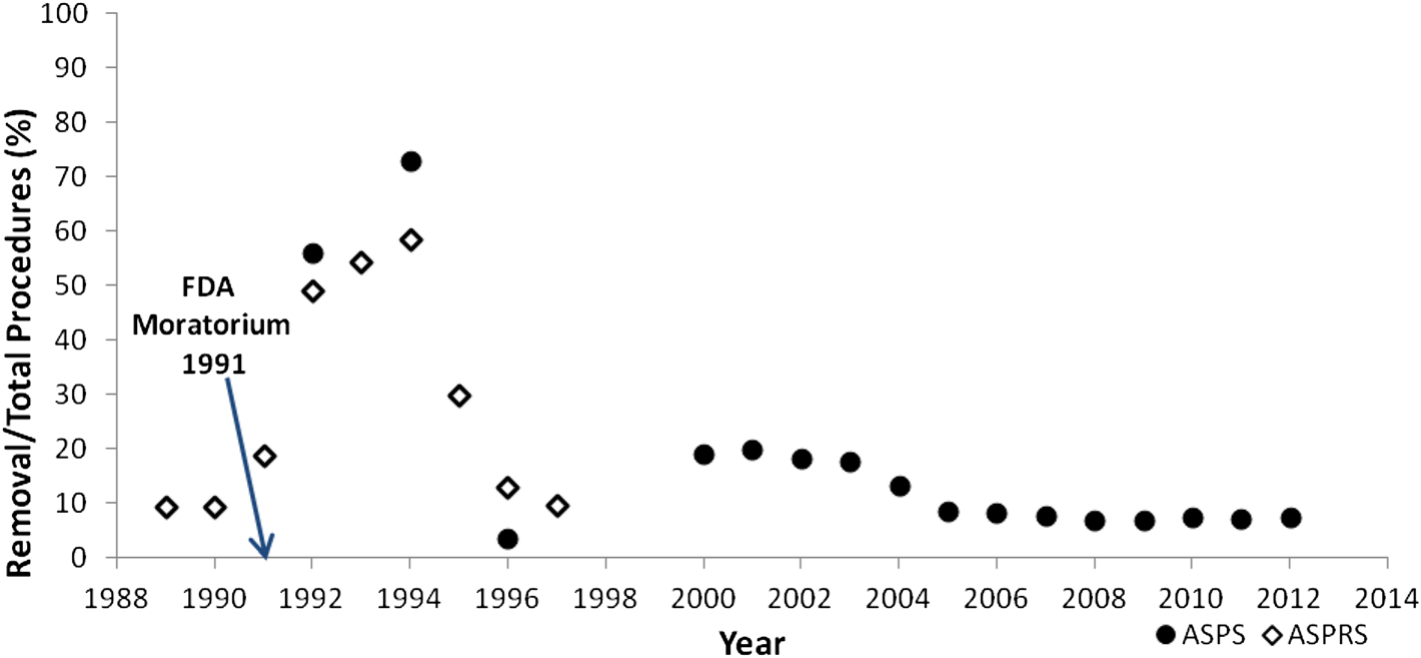
Silicone Gel-Filled Breast Implant (SGBI) explantation procedures, percent of total operations (1981-2012). Open diamonds represent SGBI explantation procedures as a percentage of total procedures from 1989 to 1997. SGBI data from 1989 to 1997 were obtained from the American Society of Reconstructive Plastic Surgeons (ASRPS), according to the Institute of Medicine’s report on the Safety of Silicone Breast Implants. Closed circles represent SGBI explantation procedures as a percentage of total procedures during 1992, 1994, 1996, and from 2000 to 2012, as reported by The American Society of Plastic Surgeons (ASPS). Arrow indicates the 1991 United States Food and Drug Administration’s (FDA) moratorium on SGBI

Although the available published studies on SGBI explanation rates only examined relatively small populations (e.g., 20-100 cases per study), historical data on explantations and total breast augmentation procedures collected by the American Society of Reconstructive Plastic Surgeons as reported by the Institute of Medicine [18] and the American Society of Plastic Surgeons [19] provides clear evidence of dramatic effects on explantation rates soon after the 1991 FDA moratorium. Data obtained in 1989 and 1990 show that out of 130,000 total procedures, approximately 12,000 removals were performed (9.2 % removal: augmentation frequency or RAF) (Fig. 2). Following the moratorium, however, removal rates increased to 18.8 % in 1991, 49.1 % in 1992, and peaked at 58.5 % in 1994. After 1994, the removal rates steadily decreased, and were at pre-1991 levels by 1997 (9.6 % RAF). The extremely high explantation rate following the 1991 FDA moratorium announcement was also reported by a subset of ASPS member surgeons for 1992 (32,607 augmentation procedures, 18,297 removals; 56.1 % RAF) and 1994 (39,247 augmentation procedures, 28,655 removals; 73.0 % RAF) (Fig. 2).

Several studies reported that patient fears were a primary reason for explantation of SGBI. Following the announcement of the FDA moratorium, The American Medical Association Council on Scientific Affairs urged physicians to recognize and address the considerable public anxiety concerning the safety of breast implants, noting that “*this anxiety is not warranted based on current scientific evidence*” [45] (Table 2). In 1995, The American College of Rheumatology issued a statement that the association between SGBI and connective tissue diseases was not proven [46]. In 1997, The American Academy of Neurology issued a statement that the association between SGBI and neurological diseases was not proven [47], and in 1998, an Independent Review Group from the United Kingdom issued a report that concluded that SGBI were not credibly associated with increased risk of cancer or with unusual connective tissues diseases [18] (Table 2). Later, in 1999, the U.S. Institute of Medicine drew similar conclusions about the weight of scientific evidence on SGBI [18]. Twelve years later in June, 2011, the FDA issued a report on the clinical trials of SGBI patients who had been monitored since the 1991 moratorium and concluded that these devices “*have a reasonable assurance of safety and effectiveness when used as labeled*” [48] (Table 2).

ASPS expanded their data collection surveys to capture a larger base of their member surgeons between 2000 and 2012 (the database rose from ~ 3000 reporting surgeons in 1996 to over 20,000 surgeons in 2000). Although ASPS [19] documented a dip in removal during 1996 (87,704 augmentation procedures, 3013 removals; 3.4 % RAF), they subsequently reported a continued wave of elevated breast implant removals from 2000 to 2004 (e.g., ~40-45,000 removals per year; ~18-20 % RAF). For this time period, ASPS [19] reported that the vast majority of explantations occurred in conjunction with new silicone-shell saline-filled implant replacement (70-89 % per year). For 2005 to 2012, ASPS [19] reported that annual rates for implant removals were lower and relatively stable (e.g., ~20-27,000 removals per year; 6.8 to 8.5 % RAF).

### Metal-on-metal (MoM) hip implants

We identified several key events surrounding the voluntary recalls of select MoM hip implants. Table 3 provides a chronological summary of selected key events and findings relating to the voluntary recalls of certain MoM hip implants and subsequent effects on their explantation and continued use on the medical device market in the U.S., U.K., and Australia.

MoM hip implants have been utilized for hip resurfacing and total hip replacement since the 1930s, although the development of a metal-on-polyethylene (MoP) implant by Charnley in the late 1950s largely replaced the early generation of MoM implants because of lower early revision rates [49, 50] (Table 3). The Charnley MoP device was found to perform well in assessments of revision rates at five and ten years post-implantation [51], but in the 1970s and 1980s, evidence showed that after about 15 years in situ*,* the polyethylene acetabular cup material more frequently disintegrated and generated plastic wear debris that caused local inflammatory responses and failure because of loosening at the femoral stem and/or the acetabular cup [52, 53] (Table 3). Despite good overall performance, the MoP hip implant could be expected to fail after about 10-15 years in service [52, 54], leading to a greater likelihood of multiple traumatic hip replacement surgeries for hip implant patients who were younger and/or more active. To help resolve this problem, a new generation of MoM implant designs was then pursued in hopes of reducing the likelihood of late failures due to material breakdown and loosening. Indeed, some patients with early generation MoM implants were reported to have good performance for more than 25 years [55–63]. In addition, more resilient high carbon content alloys and improved manufacturing techniques allowed for a new generation of MoM devices with exceptionally low rates of wear at the articulating surfaces [64–66].

The new generation of MoM hip implants were first marketed in Europe in the 1990s and showed promising results comparable to MoP devices with respect to early revision rates [49, 50] (Table 3). These newer MoMs were not allowed to be sold in the U.S. until about 2003, however, because of the more stringent FDA approval process (Table 3). A few case reports identifying unusual inflammatory responses in MoM patients were identified prior to 2008 [57, 67–70], but, subsequently, several additional reports appeared regarding early revisions due to inflammatory responses and other effects on the nervous system and the heart in MoM patients [71–75] (Table 3).

An increase of MoM hip implant recalls or market withdrawals occurred between 2008 and 2012. In 2008, the Zimmer Durom Acetabular System, an MoM design, was withdrawn from the U.S. market because of suspected higher revision rates associated with inadequate surgical procedures leading to misalignment and early revision [76] (Table 3). Between 2008 and 2011, hip implant registries in Australia and the United Kingdom separately reported observing increased rates of early revision for the DePuy Articular Surface Replacement (ASR XL) Acetabular Hip System, another MoM design that was introduced to the market in Europe in 2003 and to the U.S. market in 2005. Although there was suspicion by DePuy that inadequate surgical procedures were to blame for the increased early revision rates report for the ASR, DePuy decided to conduct a voluntary recall in Australia starting in 2009, and implemented a worldwide voluntary recall of the ASR product in 2010 (Table 3). In June of 2012, Smith and Nephew conducted a U.S. market withdrawal of the R3 Acetabular System with R3 metal liners because of reports of elevated early revision rates in conjunction with the Birmingham Hip Resurfacing System [77] (Table 3).

**Fig. 3:**
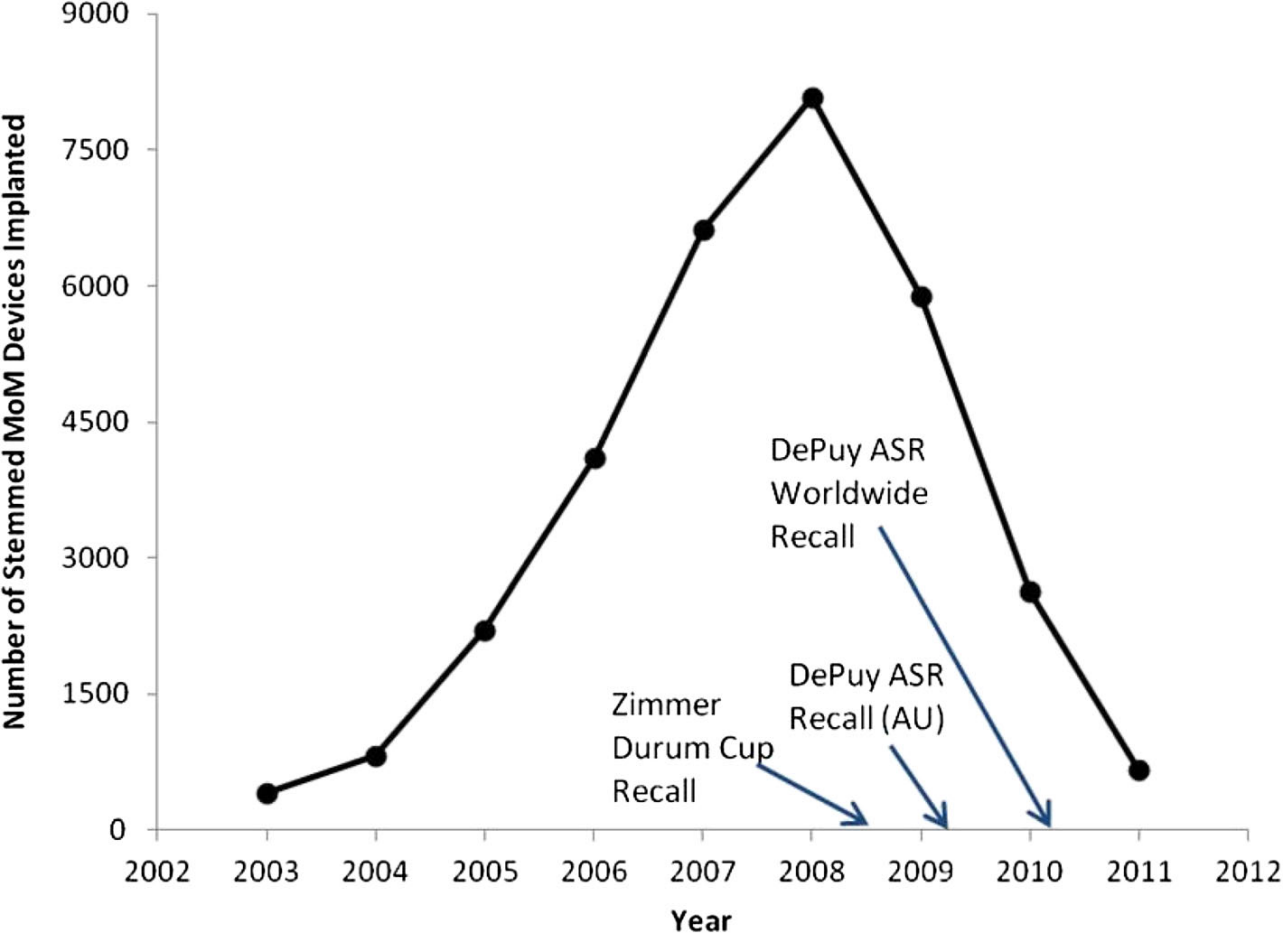
Implantations of Stemmed metal-on-metal (MOM) Devices in England and Wales (2003-2011). Based on Smith et al. [23]. Also identified are key dates of the voluntary recall of the Zimmer Durom Cup in the United States in July 2008, the Australian recall of the DePuy ASR in 2009, and the worldwide voluntary recall of the DePuy ASR in 2010

**Fig. 4:**
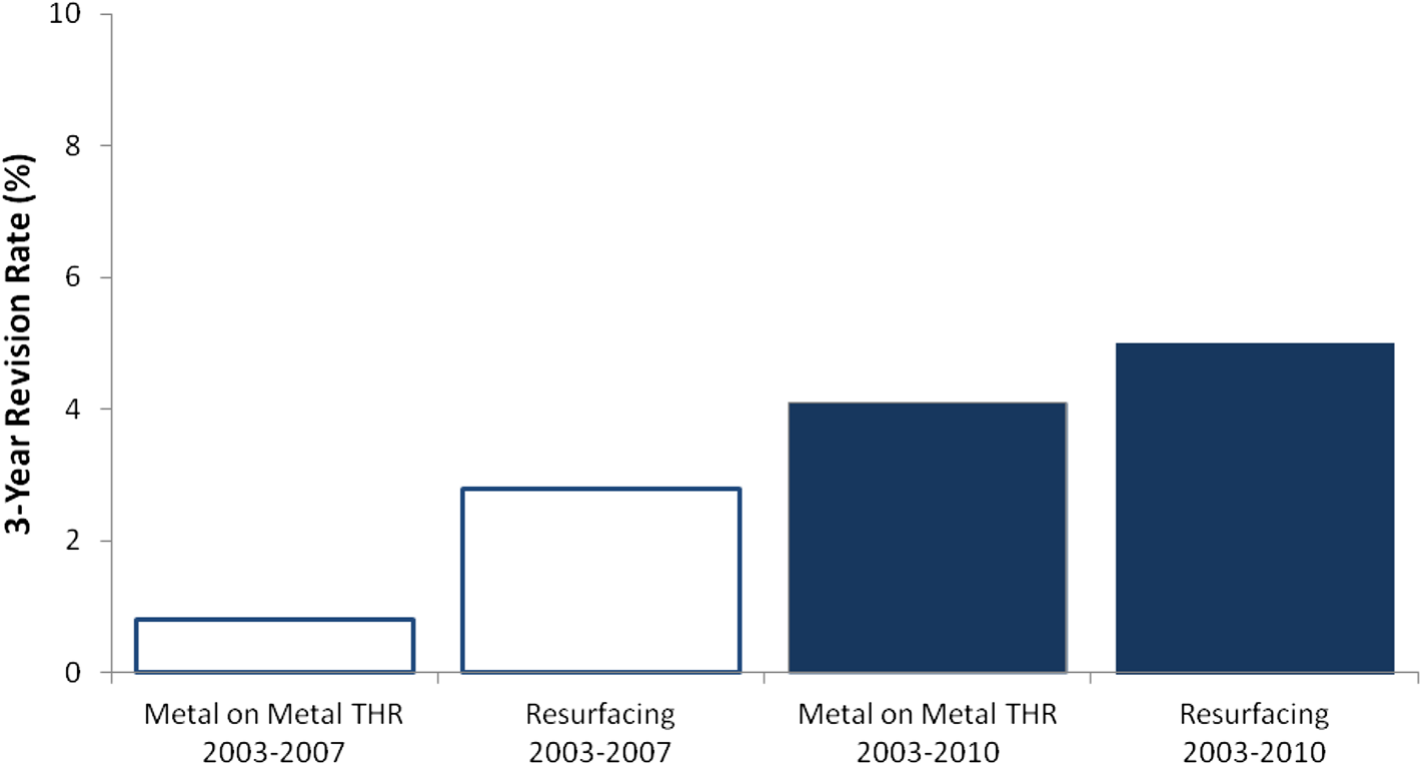
Three-year revision rates over time. The average 3-year revision rate for metal-on-metal total hip replacements (THR) and for hip resurfacing over the time periods April 2003 through September 2007 and from April 2003 through December 2010; based on results reported in the 2008 and 2011 Annual Reports of the England and Wales National Joint Registry

Figure 3 shows data on the number of MoM devices implanted in England and Wales between 2003 and 2011, identifying a rapid decline starting in 2009 when DePuy announced its first ASR recall in Australia. Despite the fact that this recall was only of a single product, use rates for all MoM devices including both hip resurfacing and total hip replacements of all device brands were affected. The voluntary recalls of these MoM devices stemmed from hip revision rate data generated by national joint registries in Australia [78] and the United Kingdom [21–23]. Figure 4 illustrates the findings on 3-year revision rates for MoM implants assessed in 2008 (monitoring patients from 2003 to 2007) and again in 2011 (monitoring patients from 2008 to 2011) showing an approximate 4-fold increase (Table 3). Comparable increases were reported for MoM patients enrolled in the Australian Joint Registry [21, 22] and in some Canadian institution-based analyses [71–73], although little data is available regarding the early revision experience in the U.S. Subsequently, negative press articles were published regarding potential dangers of MoM implants, and associated personal injury litigation ensued in the U.S. (Table 3).

### Tysabri (natalizumab)

Table 4 provides a chronological summary of selected key events and findings relating to the voluntary recall of Tysabri and effects of subsequent research and FDA advisories on recovery of the drug’s use in the U.S.

Multiple Sclerosis (MS) is a chronic inflammatory disease of the central nervous system. Patients with MS can experience debilitating physical symptoms ranging from fatigue to bladder and bowel problems and cognitive impairments involving vision, learning, and memory. Specific symptoms vary from person-to-person, but patients are generally grouped as “progressive” or “relapsing-remitting.” People with “progressive” forms of MS can experience gradually worsening problems with walking and mobility in addition to other physical and cognitive symptoms; people with “relapsing-remitting” MS (RRMS) experience cycles of exacerbations followed by periods of recovery. Tysabri (natalizumab) is a potent disease-modifying treatment for RRMS. The drug was fast-track-approved by FDA in 2004 (Table 4). At the time of its approval, Tysabri was presented as an innovative breakthrough treatment [79]. Shortly after its approval, the drug’s sponsor, Biogen, reported three cases (including one fatality) of a rare but severe and opportunistic viral infection of the brain, called progressive multifocal leukoencephalopathy (PML) (Table 4). Based on this apparent association, Tysabri was withdrawn from the market in 2005 (Table 4). Beginning in 2006, clinical trial participants were tested for PML, and safety data were gathered and reviewed by the drug sponsor and the FDA (Table 4). An FDA advisory committee studied the evidence and recommended that Tysabri be reintroduced via a restricted distribution program, “TOUCH” (TYSABRI Outreach: Unified Commitment to Health) [80] (Table 4). Since 2006, analyses of TOUCH data, clinical trial data, and continuing research have helped to clarify the risk of PML, leading to the approval of a new test to assist in PML risk stratification for Tysabri patients in 2012 (Table 4). Thus, health care providers now have a risk model to quantify and rank vulnerability to PML for patients considering Tysabri. Specifically, the presence of antibodies associated with the virus that causes PML in persons with longer duration of Tysabri treatment (>2 years) and prior use of immunosuppressant medication are known to increase risk [81]. Tysabri patients with all three known risk factors have an estimated risk of PML of 11/1000, whereas the risk of PML among other patients can range from ~1/1000 in antibody-negative persons to ~4/1000 in some antibody-positive persons [81, 82]. Risk assessment of potential Tysabri patients since 2006 has improved health and safety of this treatment regimen, including collecting baseline MRI data to help distinguish damage due to PML onset from pre-existing MS lesions, regular antibody testing, and a decline of >2 year treatment durations [82].

**Fig. 5:**
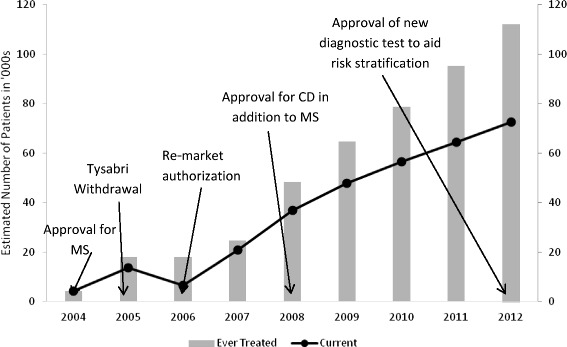
Annual and cumulative patient exposure to Tysabri (2004-2012). Data are from Elan Corporation or Biogen Idec Inc. Fourth Quarter Reports for each calendar year and are based on information from the TYSABRI Outreach: Unified Commitment to Health (TOUCH) prescribing program database and other third party sources. Arrows identify key dates of FDA initial approval (November 23, 2004), withdrawal (February 28, 2005), re-market authorization under the TOUCH restricted distribution program (June 5, 2006), FDA approval of Tysabri for Crohn’s Disease (January 14, 2008), and approval of a new test to assist in PML risk stratification for Tysabri

Before the withdrawal of Tysabri in 2005, approximately 18,000 patients had received Tysabri, including 4300 patients during clinical trials and an additional 13,700 patients post-marketing (Fig. 5). After reintroduction to the prescription drug market in 2006, patient enrollment and sales of Tysabri became more uncertain and slowed considerably relative to initial expectations for the drug [83] (Fig. 5). Even after Tysabri was approved for Crohn’s Disease on January 14, 2008, and predictions were adjusted, new patient growth continued to be slower than predicted [31, 84]. The number of patients in 2010 was less than 80,000, well short of the 100,000 patient expectation announced by Biogen post-PML in 2008.

## Discussion

We defined the four cases above as “highly beneficial” based on several lines of evidence: 1) in the case of the Dalkon Shield IUD, the peer-reviewed literature supported the efficacy of and patient satisfaction with the IUD [16, 34, 36]; 2) SGBI had been estimated to be used by nearly one million women [37], demonstrated relatively low rate of rupture-related device failures and few reported serious clinical consequences through the late 1980s [43], and had been declared by the FDA, in 2011, as reasonably safe and effective based on clinical monitoring [48]; 3) MoM hip implants were reported to have good performance for more than 25 years [55–63], and exceptionally low rates of wear at the articulating surfaces [64–66]; and 4) Tysabri benefits outweighed the risk of PML in patients with appropriate MRI and antibody screening results [85]. Based on our review of the use data and documentation of patient and public sentiments surrounding each device or drug, we determined that these examples illustrated overgeneralizations, such as a negative halo effect and spillover effect, and we found that certain common factors appear to affect all four examples of consumer usage of the medical devices and drugs.

### Patient fear

A negative halo effect may be driven predominantly by patient anxiety rather than by adverse clinical outcomes. The consequences of patient fear were apparent for all three of the implanted medical device examples, since the rate of more serious adverse health impacts from each device was low, while the rate of explantation soon after the stigma was propagated was high. Similarly, in the case of Tysabri, even with a risk assessment system that can predict adverse clinical outcomes, patients and physicians may decide to discontinue using Tysabri due to patient concerns, even when the benefits of treatment appear to outweigh the risks.

The high rates of SGBI patient requests for implant removal in the U.S. were studied in some detail during the 1990s, and were largely attributed to patient fears rather than unexpected clinical consequences of the device. Rohrich et al. [86] reported that 282 of 720 patients (29.2 %) referred for possible SGBI removal between December, 1992 and January, 1996 underwent explantation; the most common reasons for explantation were “*ruptured implants, severe or recurrent capsular contractures, and desire for elective removal based upon continued concern about silicone gel-filled implants*”. Slavin and Goldwyn [87] studied 46 SGBI patients who underwent explantation and reported that 45 % of them said that fears based on media reports had driven their decision. However, 74 % of these explantation patients had their SGBI devices replaced with saline-filled (silicone shell) implants that might pose similar risks associated with the device shell [87]. Similar findings were reported in several other small studies of SGBI explantation study groups in the 1990s [86, 88–92]. Some researchers have suggested that patients with depression and other neurologic diseases (e.g., somatization, obsessive-compulsiveness, hostility, and anxiety) were over-represented among those seeking SGBI explantation [88, 89, 92], and among those reporting more numerous subjective complaints [93]. However, significant patient fear of implant-related health problems, regardless of depression or other mental health issues, is considered an adequate clinical indication for SGBI removal or replacement [94]. The data demonstrate an initial rise in SGBI removal frequency immediately after the FDA moratorium that may represent the most fearful individuals among the large population of SGBI patients at the time. With the continued negative press and publicity due to mass tort litigation regarding these implants through the late 1990s, the elevated rate of SGBI removals apparently continued for more than a decade, and then fell to a lower, stable rate starting in 2005 (Fig. 2).

Patient and physician anxiety may have also played a role in decisions to remove MoM hip implants. With the three voluntary MoM hip implant device recalls in place, surgeons were likely to recommend alternative, non-MoM devices to avoid a possible repeat of early revision, even if their track record with these devices had been favorable. Perhaps more important is the potential effect of these recalls on decisions to explant MoM devices in the absence of clinical signs that would normally be required to support a recommendation of revision (e.g., clinically important periprosthetic inflammation, pain, and/or limited range of motion). As seen with SGBI following the FDA moratorium, patients or physicians might reasonably perceive that a voluntary product recall suggests there is sufficient reason to remove the device as a result of patient fears alone. Thus, assessment of a patient’s subjective fears may in some cases supersede an individual-specific analysis of the risk versus benefit based on objective clinical signs. However, the potentially complicated and traumatic surgical procedures that may be involved with hip revision surgery must be carefully considered.

There could be other explanations for increased revision rates of MoM hip implants. The second generation MoM hip implants generated lower volumes of wear debris than the more common metal-on-polyethylene (MoP) implants that typically failed after a 10- to 15-year useful lifespan [52, 54, 64–66, 95]. Hence, MoM hip implants could be used in younger and more active individuals in hopes of longer useful lifespans, since reports have identified first generation MoM implant survival with good function beyond 25 years [55–63, 96]. This durability aspect may correspond to a broader set of patient characteristics and implant performance demands. For example, a greater fraction of younger patients could increase the population with congenital hip dysplasia or traumatic injuries leading to an increased potential for implant failure when compared to osteoarthritis patient populations. In addition, assurances of improved durability could also lead younger or more active patients to exceed limitations on activities that increase implant wear and/or risk of re-injury of the hip joint, with the potential for an increased risk of implant failure beyond that expected for older osteoarthritis patients. Thus, the unique demographics of the MoM population may have led to increased numbers of revision surgeries. Additionally, in younger/healthy patients, the risk of harm may not be sufficient to warrant revision surgery based on fears alone.

It should also be noted that Reito et al. [97] reported that “*indications for revision surgery can vary greatly between different surgeons and different hospitals. Some surgeons may prefer closer follow-up in cases where others would prefer revision surgery. The current literature lacks a specific definition for adverse reaction to metal debris (ARMD) and especially the indications for revision. Due to these implant and inter-observer related differences, a high heterogeneity is observed.*” The extent to which physician or patient fear also contribute to this heterogeneity is unclear [97].

Patient and physician fear of previous risks may have also impacted the effects on Tysabri use. After Tysabri was placed back on the market and warnings of PML risk were added, RRMS patients were caught in a quandary. Patients who were not yet on Tysabri had to weigh the risk of worsening MS versus the risk of acquiring PML. Those patients who were already on Tysabri and who had experienced significant quality of life improvements were faced with the fact that the risk of acquiring PML was increasing with duration of treatment. Interviews of Tysabri patients in 2009 suggested that even after treatment had begun and general fears associated with Tysabri treatment abated, a heightened uncertainty about PML continued [98]. Patients chose treatments in consultation with their physicians and, at a time when there were few alternative treatments of similar potency available, some patients were willing to accept greater risks than their physicians would consider acceptable. Despite the fact that patients and physicians today have a similar appreciation of the risks of PML, physicians are more risk averse than their patients about Tysabri, possibly because of concerns about patient safety and risk of mal-practice litigation [99, 100].

### Spillover effect

Similar to Vioxx [11], the negative halo effect generated by negative reports on one particular device can create a spillover effect, in which the stigma rapidly spreads to affect use patterns for arguably dissimilar products in the same category. This phenomenon clearly occurred in the U.S. following withdrawal of the Dalkon Shield and MoM hip implants, as reflected in decreased usage across all devices of a category as a result of the negative reports and mass litigation.

Following the removal of the Dalkon Shield from the market in 1974, the use of all IUDs began to decline, even though numerous IUDs remained on the U.S. market, including the Copper 7 and the Tatum T, which were introduced in 1974 and 1978, respectively [16]. Similarly, reports of increased early revision rates for certain MoM products between about 2008 and 2011 led three separate manufacturers to remove the potentially problematic devices from the market in the U.S. and elsewhere while other popular MoM products remained on the market. Subsequently, numerous negative press articles were published regarding potential dangers of MoM implants, and associated personal injury litigation ensued in the U.S. involving thousands of individual cases. The voluntary withdrawal of the three hip implant devices and associated negative press reports led to dramatic declines in physician use of MoM devices generally, likely reflecting a stigma effect on surgeons as well as their hip implant patients, comparable to the decline in IUD use after the Dalkon Shield withdrawal (Fig. 1). Despite the fact that this recall was only of a single product, the stigma appeared to affect use rates for all MoM devices including both hip resurfacing and total hip replacements of all device brands (Fig. 3).

The effect of stigma on Tysabri use as a treatment for Multiple Sclerosis and Crohn’s Disease differs from the negative halo effect seen for the medical device examples and for Vioxx and similar drugs. Tysabri is one drug among a relatively small number of available treatment options for these diseases, and may exhibit potentially severe side effects and inconsistent efficacy for each. Its continued use today in conjunction with more detailed warnings and medical screening to limit the risk of severe side effects is perhaps a reflection of its smaller patient population and limited number of similarly efficacious treatments for these particular diseases. In contrast, a wide variety of anti-inflammatory drugs of arguably similar efficacy and more limited side effects were available to replace Vioxx. Moreover, the large patient population taking Vioxx afforded more opportunities to identify and recruit a large and diverse plaintiff population for profitable and sustained campaigns in personal injury litigation against the makers of Vioxx in the U.S.

### Uncertain scientific findings

Third, a negative halo effect on an entire category of medical devices can be sustained regardless of the scientific findings pertaining to safety. Patient anxiety generated by uncertainties often relate to conflicting or incomplete scientific evidence. In regard to the Dalkon Shield IUD, increased FDA regulation, negative press reports, and litigation concerning the Dalkon Shield prompted further investigations of all IUDs during the 1970s and 1980s. These studies suggested that using IUDs generally contributed to higher levels of pelvic inflammatory disease (PID) in women [36]. Although the initial studies reported that all IUDs, regardless of design differences, increased the risk for PID, other IUD designs were later shown not to be as problematic as the Dalkon Shield. Thus, the risk for PID in other IUDs was reported to have been overestimated [36]. Cheng [16] summarized numerous studies indicating that the risk for PID was no higher in non-Dalkon Shield-IUD users who were at a low risk for sexually transmitted diseases (STDs) than for those using no contraception. In fact, when studies were adjusted for confounding variables such as sexual behavior, IUD use was not found to be predictive of PID [101]. Furthermore, misconceptions regarding using IUDs as an abortifacient and their role in infertility were prevalent [101].

However, as noted by Hubacher [101], even when considering data to the contrary, the stigma regarding the IUD was difficult to overcome. He explained: *“Even weak research that supports these arguments can appear to make the claims irrefutable. Research that generates results counter to these assumptions has to be flawless, unchallengeable and repeated to have any lasting impact. Thus, although the most recent research has shown these assumptions to be unwarranted, the controversy continues”* [101]. Thus, though the design flaw of the Dalkon Shield was markedly different from other IUDs, assumptions regarding research studies that grouped all IUDs together altered women’s perceptions and led to lower rates of IUD use for decades.

Additionally, Forrest [34] reported on a survey conducted in 1991 that examined the method by which women received their information regarding IUDs. Although 52 % of women received information from their doctor or doctor’s office, nearly 25 % of women received primary information from magazines, books, advertising, or educational TV, and another 19 % received information from friends and relatives [34]. In fact, younger women and Hispanic women born outside the U.S. were reported to be more likely to be interested in using an IUD versus older women and women born in the U.S. or Puerto Rico [34]. Based on these statistics, one can hypothesize that the stigma created from the negative publicity regarding the Dalkon Shield had a greater effect on exposure to the negative information, which was more broadly publicized in the U.S. than in other countries [102]. The uncertain scientific findings regarding various studied IUDs, negative reports from the Dalkon Shield incident, and the resulting voluntary removal of the Dalkon Shield from the market, had both immediate and lasting effects on women’s aversion to choosing IUDs as their method of contraception (Table 1, Fig. 1), despite their proven safety and efficacy [15, 16, 36, 101].

Similar to the IUD saga, uncertain scientific findings plagued the reputation of SGBIs as a product type, regardless of design features or manufacturer. Excepting the common occurrence of capsular contracture, SGBIs were presumed to be reasonably safe through the late 1980s given their relatively low rate of rupture-related device failures and few reported serious clinical consequences [43]. Furthermore, capsular contractures are not unique to SGBIs, and have been shown to also occur with saline-filled silicone shell implants that ultimately replaced SGBIs in the medical device market [39]. Furthermore, reported rupture rates by Brown et al. may have been overstated since all degrees of implant collapse based on radiologic assessment were grouped together (i.e., including subclinical changes) and the majority of these asymptomatic patients had their SGBI implants for more than 15 years [42]. It is clear that breast implants cannot be reasonably expected to last for a lifetime. Indeed, the need for explantation and/or replacement of breast implants due to leakage or collapse after many years in situ can be expected due to the commonality of contracture and potential implant trauma related to procedures for release of contracture.

The FDA moratorium decision on SGBI occurred after a CBS television show in 1990 (“Face to Face with Connie Chung”) had sensationalized the concerns about breast implants being associated with autoimmune disease, blaming FDA for permitting hazardous medical devices to be sold [43]. In 1991, when FDA decided to limit access to SGBIs, SGBIs had not been recalled by their manufacturers, and had been in use for nearly three decades for elective breast augmentation and reconstruction without reports of excessive rates of rupture or associated clinical disease. Yet a formalized monitoring program to evaluate SGBI patients was not in place to provide convincing data on their safety and/or implant longevity. Furthermore, despite the litigation-related claims of SGBI-associated connective tissue disease, no epidemiological studies regarding connective tissue disease in women with SGBIs had been performed prior to 1994 [43]. Later, the scientific evidence for hypothesized associations between SGBI and certain disease states such as connective tissue diseases, breast cancer, and neurologic complaints was evaluated by a variety of medical authorities over the prior two decades, with the uniform conclusion being that these associations were very weak and not well validated. Thus, in general, it appears that published medical advice in statements from medical authorities regarding the tenuous nature of scientific evidence for SGBI-disease associations was not heeded by U.S. patients or plastic surgeons through the 1990s and early 2000s.

Angell [43] and others [44, 90, 103, 104] have noted that sensational media reports, combined with even an ambiguous endorsement of concern by FDA, can lead to stigma that is nearly impossible to undo, even after the weight of scientific evidence has become clear. Handel et al. [44] reported that “*negative media publicity may cause unnecessary anxiety, depression and remorse*” and lead to decreased patient satisfaction and ~5-fold greater frequency of implant removal requests when comparing patient views before versus after the FDA moratorium. Handel et al. [44] also noted that while satisfaction is determined by whether the patient herself has experienced complications (like implant rupture or capsular contracture issues), “*our study discloses that media reports have a major effect on patient concern*.” The SGBI story illustrates the role that media reports can play in presenting tenuous scientific information as known facts and thereby increasing patient fears that can trigger high rates of device removal following the 1991 FDA moratorium. Notably, the most prominent problem with SGBI (capsular contracture) was not resolved with the subsequent generation of saline-filled, silicone shell breast implants. Moreover, there was a relatively low rate of SGBI failure due to implant rupture (~5 %), so the clinical benefits of explantation were absent in the vast majority of cases, other than helping to resolve anxiety from the stigma on SGBI induced by the 1991 FDA moratorium. With an estimated one million U.S. women having these breast implants as of the early 1990s [43], the stigma from the FDA moratorium and associated news reports [44] and litigation generated patient uncertainty, and likely unnecessary explantation surgeries in the absence of strong or consistent scientific support.

As with SGBI, some authors have commented on publication bias regarding failure and explantation of MoM hip implants, particularly regarding the Articular Surface Replacement XL Total Hip Arthroplasty device (ASR XL THA) that was subject to a worldwide manufacturer recall and multi-district litigation in the United States. Reito and colleagues [97] recently examined the prevalence of MoM hip implant failure due to adverse reaction to metal debris (ARMD) including a systematic literature review and meta regression analysis. These authors reported that publication bias may have affected available scientific studies regarding potential health effects from MoM hip implants in that the year of publication was an important variable to consider as a confounder in their analysis. The authors noted that,

“*there has been considerable publication bias in the MoM literature during recent years. As a result, there has been a strong tendency to publish as high as possible prevalences of pseudotumors and ARMD.* […] *Moreover, prior to 2010, MoM hip replacements were popular and there was a trend toward positive results instead. The trend towards positive results can be observed in the numerous studies that report favorable results with the Birmingham Hip Resurfacing (BHR) device. Furthermore, to support this statement, there are no studies prior to 2010 that have reported, for example, the results of the ASR XL THR, which was eventually shown to have been disastrous”*

As noted earlier, the lack of a clear definition for ARMD and/or general agreement among hip surgeons on objective criteria for ARMD-related revision surgery has made meta-regression analysis difficult to interpret [97]. Thus, widespread publication of heterogeneous and arguably equivocal scientific findings regarding MoM-related ARMD is influenced by the clearly unfavorable findings for the ASR XL THA device and may have created a high potential for spillover of stigma across all MoM hip implants.

Furthermore, litigation pressure related to defective product/personal injury claims publicized by legal counsel and news media in the U.S. can lead to sustained stigma on an entire category of medical devices regardless of how limited or tenuous the scientific findings might be on a given product. This influence has clearly affected use and explantation rates for IUDs and SGBI, and will likely occur with MoM hip implants going forward. And in the case of SGBI, the replacement (saline implant) product was comprised of a silicone shell that likely posed similar risks for the most prominent clinical problem (contracture) associated with SGBIs. Notably, after two decades of clinical trials, FDA determined that SGBIs were reasonably safe in 2011 [48]. In regard to Tysabri, patients often discontinue Tysabri as they approach 24 months of treatment, even though risk analyses show continuing treatment may be prudent, as the benefits of Tysabri outweigh the risk of PML in patients with appropriate MRI and antibody screening results [85]. Moreover, demonstrably superior MS treatment options have yet to emerge, and, for some patients, Tysabri may be the most viable treatment option in terms of efficacy and/or risk of serious side effects [105–107]. The Tysabri case brings up numerous questions: what is the degree to which uncertainty and fear contribute to treatment decisions? How much are treatment decisions impacted by a state-of-the-art understanding of risks versus risk communication and risk comprehension? A technical characterization of PML risks clearly may not be in and of itself enough to address the fear and uncertainty the average patient feels.

### Persistence of negative halo effect

Recovery of a product’s safety reputation and prevalent use may take decades in the U.S., even while these products may exhibit widespread use and good safety records in other countries. The recovery of contraceptive market share for IUDs in the U.S. market took decades to recover, even while IUD usage in Europe and other countries remained higher and consistent. Negative publicity surrounding the Dalkon Shield influenced IUD use in the U.S. for decades, as evidenced by the drop in favorable opinions from 1972 to 1994, as well as the precipitous drop in IUD use and slow recovery in the early 2000s (Table 1). Interestingly, though fewer women in the U.S. utilized IUDs when compared to other methods of contraception, Forrest reported that users were “highly satisfied” with them [34]. In fact, the efficacy of IUDs in preventing pregnancy is reported to be comparable to surgical sterilization, and the overall costs to the patient are lower than those for other contraceptive methods [16, 36].

With over 100 million contraceptors using IUDs worldwide, this method is reportedly used by more women than any other reversible method of birth control [16, 36]. Presently, only hormone delivery devices and the copper ParaGard device are used in the U.S., despite the fact that several other options are used in Europe [16, 101, 108]. Sonfield [35] reported that only 5.5 % of contraceptors in the U.S. use an IUD, as opposed to up to 27 % of contraceptors in European nations, such as Norway. Moreover, Hubacher [101] reported that 21 % of contraceptors in Mexico use an IUD. Thus, the stigma created from the Dalkon Shield saga not only affected the product itself, but had a lasting impact (decades long) on the prevalence of IUD use generally in the U.S.

Interestingly, rates of IUD use are increasing in the U.S. within the past decade, probably because of the maturation of a new consumer group and a growing number of medical practitioners who were not practicing professionals at the height of the Dalkon Shield saga. Thus, the stigma appears to be fading. However, according to Sonfield [35], the stigma of the Dalkon Shield had “*a lingering, indirect impact.”* Because of the stigma regarding IUDs, few doctors have been trained in IUD implantation in the U.S. because so few patients have used these devices over the last few decades [33]. Now, when the stigma regarding IUDs appears to be diminishing, access to the IUD may be limited even for women who choose it, due to the lack of U.S. practitioners who are trained in the insertion and prescription of the IUD [33].

As seen following the 1991 FDA moratorium on SGBI, a stigma-related increase in explantation procedures can reasonably be expected to be seen as a result of the negative press reports and massive U.S. litigation regarding the recalled MoM devices. The rate of SGBI revision due to rupture was only about 5 %, yet a sustained increase in explantation rates was documented over time that was more likely generated by fears as opposed to objective clinical outcomes (Fig. 2). For MoM implants, the rate of early revision related to severe local inflammatory responses appears to be on the order of 5 to 10 %, although a few higher estimates have been reported. Like the capsular contracture response commonly seen in SGBI and other breast implant patients, the local tissue inflammatory responses in MoM patients occur over a broad spectrum of severity, and are affected by subjective tolerance factors; thus, rates of clinically necessary revisions may be difficult to discern. Given the extensive U.S. litigation and negative publicity regarding MoM implants in the past few years, decisions on device explantation likely will be influenced by recall-related stigma, and elevated revision rates will be observed for many years to come.

The emergence of alternative therapies combined with Tysabri’s history present a kind of inertia that is not so easily overcome. Both physicians and patients know more about the risk of PML to Tysabri patients today than they did in 2005. Nevertheless, the growth in the number of patients on Tysabri remains well short of expectations.

The four examples cited above illustrate that negative news media reports and defective product/personal injury litigation regarding the potential health hazards posed by medical devices and drugs can greatly impact use rates in the U.S. market. Such negative impacts on use patterns of medical treatments may occur even when the scientific support for such dangers is tenuous, and may be sustained for many years due to the often protracted nature of personal injury litigation. This negative halo effect has affected many drugs and medical devices that were withdrawn from the market for different reasons, and also affects products that were never directly implicated as having safety issues.

### Study limitations

This analysis has several limitations. Given that this was a retrospective analysis of complex phenomena involving mixed social roles (e.g. patient, physician, consumer), the testing of our hypothesis regarding the impact of overgeneralizations on patient perceptions and decisions were often unquantifiable and partially dependent on limited data sets collected around the time of the recalls. Furthermore, the selected examples were identified in part by the volume of available data and published information and are not based on a systematic search and content analysis of all potentially relevant media reports or on the collection of new survey data; hence, they highlight more controversial decisions that other researchers have chosen to comment upon. Also, there are many social and cultural aspects that can influence medical decisions at different institutions (e.g., government agencies or healthcare providers or insurers) and at different levels of perceived authority (e.g., regulators or researchers or physicians), and this analysis only touches upon limited aspects. Additionally, risk perception by patients and physicians and associated stigma-related actions are complex social and psychological phenomena that have been discussed only briefly. We aimed to compensate for these potential limitations in our methodology, which included a multifaceted description of each recall or withdrawal case, including analysis of usage data, peer reviewed literature regarding product efficacy, product safety, consumer perceptions of products over time, and changes in regulation in order to identify the presence of overgeneralizations, such as the halo effect and spillover effect, and the impact they had on product use, public health decisions, and policy shifts. The analysis of these cases demonstrates that these overgeneralizations may have contributed to the decline of a product’s usage and impacts on public health.

### Study implications

This analysis increases our understanding of the multitude of factors that impact the use of beneficial medical devices or drugs and can help us make decisions to identify and mitigate overgeneralizations that may have negative impacts on public health. For example, it may be possible to prevent or mitigate the types of overgeneralizations described here. However, mere “education” is not enough; since overgeneralizations are unconscious and automatically applied, simply knowing more may not be helpful to a layperson unless those persons become experts with respect to the risks they face. Interestingly, formal risk analytic processes were successfully developed for Tysabri once the PML risk factors became clearer. The adoption of formal decision analytic tools by physicians helped both physicians and their patients [109]. Additionally, improving awareness of the fact that we are subject to biases may help. For example, drawing patients or consumer attention to the halo effect may mitigate the consequences of the halo effect [110].

As risk perception has social and cultural components, there is a limit to what can be accomplished at the individual person level. To reduce a negative halo effect leading to a withdrawal or recall event, or following an event, manufacturers and regulators must work closely together to develop strategic approaches to risk communication. Notably, all three implant devices reviewed in this manuscript could be interpreted as being ‘grandfathered-in’ (e.g., by pre-regulation usage and/or being considered substantially similar to a previously tested/approved device) under the concurrent FDA regulatory approval system without receiving sufficient safety testing. However, opposing interests might argue that ongoing patient monitoring during successful clinical use of these devices over decades should be carefully considered as proof of safety. In that respect, one of the lessons learned from these cases is that product manufacturers must more broadly understand their product. More specifically, after FDA approval, manufacturers should plan on implementing a formalized and adaptable monitoring program that obtains objective data to address any health concerns that may arise. This program should characterize the nature and magnitude of the potential health risk and communicate any such information to current and future patient consumers and their physicians. Product manufacturers must be prepared to answer difficult questions (e.g., factors affecting implant durability/longevity or unrecognized disease associations) with data generated through ongoing monitoring of the patient consumers over time.

The quality of reporting of scientific findings and uncertainty regarding scientific findings and negative outcomes heavily influences negative media and consumer behavior [111]. According to Nelkin (1995), risk reporting can be sensational, confused, and misinformed [111]. The media rely on available information from sources that can perpetuate confusion during rapidly unfolding events [111]. An example of this scenario was recently reported on by Matthews et al. [112], in which the authors documented a decrease in statin use by current users following a high media coverage period. The authors described the coverage as an “intense” public discussion over the risks-benefit balance of statins. Such widely covered health stories can lead to a lack of clarity and have the potential to impact healthcare related behavior [112]. Thus, product manufacturers should attend closely to the emergence and spread of powerful narratives driven by uncertainty and fear of side effects. It is important to note impacts on both patient and physician behavior.

Furthermore, product manufacturers, public health officials, and regulators should acknowledge patient and physician fears as motivating factors for device removal or discontinuation of a product. By recognizing the motivating role of fear, these stakeholders may determine methods for providing objective product information and risk-benefit analyses specific to each patient. Physicians must also acknowledge patient fear and psychosomatic symptoms when considering motivation for removing medical devices in order to understand the extent of a patient’s knowledge regarding the product’s safety, provide medical advice, and also inform the patient regarding the risks and benefits of removal procedures. Likewise, product manufacturers must not underestimate the role that physician concerns play in attending to and reacting to patient fear, or the role that litigation plays in controlling a physician’s decision to recommend alternative devices. When possible, researchers should control for patient and physician fear in future epidemiology studies when determining reasons for implant removal or choice of product.

The negative impressions generating the halo effect in each case often were not empirically derived, and therefore were resistant to newly discovered information and facts. This negative halo effect relating to stigma is most prominent in the U.S. as a result of its adversarial legal system [43]. Indeed, the environment in which science cannot play a role in changing beliefs and attitudes attracts litigation because non-scientific grounds (e.g., company negligence or fraud claims) are associated with higher financial stakes. Litigation and regulatory decisions in the U.S. have clearly played substantial roles in generating stigma that ultimately affects use patterns of entire categories of beneficial drugs and medical devices, with potential lasting impacts on public health and policy. Going forward, public health stakeholders and policy makers must consider that reported increases in device failure and explantation following specific device recalls or withdrawals may be driven mostly by patient fears rather than by clinically important adverse effects.

Historical cases indicate that withdrawal events can affect the use patterns of similar, but safe products because of both consumer and physician concerns of safety or litigation risk. Product manufacturers must anticipate this outcome and have a communication framework in place to minimize non-evidence based rejection of their product. They must understand product usage and removal rates, as well as understand comparisons to similar or dissimilar products in the same category. Accurate tracking of patient use, removal, and physician prescription can help indicate early patterns of decline associated with other product withdrawals or recalls.

In the case of Tysabri, a risk factor model was developed and was able to provide information for physicians and patients regarding product prescription; however, patient fear and stigma still played a major role in patient choices. Thus, product manufacturers must understand how consumers receive information regarding their products. In the case of the IUD, only half of women received information from physicians, and the other half sought information from popular press, friends, or family [34]. We caution that researchers should consider the sources of information feeding overgeneralizations when assessing the medical implications of higher revision rates following product recalls and withdrawals. Consideration of recall-related stigma should be understood by patients and surgeons alike so that clinically objective risks are not overstated, and so that the risk versus benefit decisions of pharmaceutical discontinuation or medical device removal are based on objective scientific data and are not skewed by unwarranted anxiety.

Differences in cultural factors between the U.S. and European countries may be expected to influence the halo and spillover effects described in our four cases, although such factors can only be assessed qualitatively. Differences between U.K. and U.S. systems of medical care and medical insurance are one example, where the infrastructure of the U.K. national healthcare system can be expected to simplify and streamline certain choices (e.g., approved drugs or devices) that in the U.S. are subject to influences from more diverse stakeholders (e.g., drug or device manufacturers, private insurance companies, medical organizations, etc.). The legal system differences are another potentially important difference, where medical and scientific experts in the U.K. system are advisors to the Court while the U.S. system is advocacy-based and injects the ‘battle of experts’ into legal decision making. Also, sensational journalism targeting alleged misdeeds or inadequacies of regulatory officials, corporate entities, physicians, testifying experts, and even judges is more rampant in the U.S. when compared to the U.K. These advocacy-based cultural factors in the U.S. may promote greater distrust of medical and scientific authorities by patients and citizens generally, and undoubtedly affects the nature, volume, and outcomes of U.S. personal injury litigation compared to the U.K.

Finally, it is important that this paper not be seen to be supporting flawed risk perception as a cudgel for blaming the victim [113]. By highlighting kinds of overgeneralizations, such as halo and spillover effects, we hope to leverage greater investment by regulators and manufacturers in risk communication. Ultimately, we believe that improvements in the messages to physicians and the general public surrounding recalls and withdrawals could help to prevent or mitigate the kinds of faulty reasoning which may lead to unintended and negative health care decisions.

## Conclusions

Since this research was completed, additional examples have come to light, the New York Times recently reported that litigation and fear of medication side effects may have influenced physician and patient behavior resulting in otherwise avoidable osteopathic fractures [114]. We have proposed a mechanism to explain these phenomena and a possible approach to counteract the effects. In sum, we conclude that the ‘negative halo effect’ associated with a stigma -- rather than an objective risk-benefit assessment of medical products -- can increase negative health outcomes for patients due to reduced or inappropriate product usage. The four examples examined in detail here provide a historical perspective spanning from the 1960s to present and were selected in part based on the scientific and social controversies raised by the influence of equivocal scientific evidence, regulatory actions, product recalls, and/or related litigation. For all drugs and medical devices, it is crucial for manufacturers and the medical community to have adequate aftermarket surveillance mechanisms in place and to promptly and rigorously evaluate adverse outcomes. The risk-benefit considerations must be clearly and transparently communicated to patients and physicians, including the associated strengths weaknesses and uncertainties of the scientific findings. Unwarranted negative halo effects and spillover of stigma in the absence of reasonably rigorous scientific proof can sometimes lead to serious negative consequences in terms of treatment choices and quality of life for patients in greatest need.

## Abbreviations

ASRPS: American society of reconstructive plastic surgeons
ASPS: American society of plastic surgeons
ASR: Articular surface replacement
FDA: Food and drug administration
IOM: Institute of medicine
IUD: Intrauterine device
MoM: Metal-on-metal
MoP: Metal-on-polyethylene
MS: Multiple sclerosis
NJR: National joint registry
PID: Pelvic inflammatory disease
PML: Progressive multifocal leukoencephalopathy
RAF: Removal: augmentation frequency
RRMS: Relapsing-remitting multiple sclerosis
SGBI: Silicone gel-filled breast implant
STD: Sexually transmitted disease
TOUCH: Tysabri outreach unified commitment to health.
